# Use of *Spilopelia senegalensis* as a Biomonitor of Heavy Metal Contamination from Mining Activities in Riyadh (Saudi Arabia)

**DOI:** 10.3390/ani9121046

**Published:** 2019-11-29

**Authors:** Ahmed M. Almalki, Jamaan Ajarem, Ahmed A. Allam, Hamed A. El-Serehy, Saleh N. Maodaa, Ayman M. Mahmoud

**Affiliations:** 1 Zoology Department, College of Science, King Saud University, Riyadh 11451, Saudi Arabia; Ah797979@hotmail.com (A.M.A.); helserehy@ksu.edu.sa (H.A.E.-S.); maodaa_28@yahoo.com (S.N.M.); 2 Zoology Department, Faculty of Science, Beni-Suef University, Beni-Suef 62514, Egypt; allam1081981@yahoo.com; 3 Oceanography Department, College of Science, Port Said University, Port Said 42522, Egypt

**Keywords:** heavy metals, pollution, biomonitoring, mining, oxidative stress

## Abstract

**Simple Summary:**

Bioindicators and biomonitors are living organisms utilized to appraise the health of the environment or natural ecosystem. Mining, which refers to extraction of valuable materials from the earth, represents a source of heavy metals that can impact the environment, biodiversity, and human health. We investigated the value of the laughing dove (*Spilopelia senegalensis*) as a biomonitor of environmental contamination with heavy metals from mining practices. Our results revealed the accumulation of heavy metals in the liver, kidneys, and lungs of the laughing dove collected from the mining site. The doves exhibited tissue dysfunction and injury, and decreased antioxidants. These results show the value of the laughing dove as a biomonitor of environmental pollution with heavy metals.

**Abstract:**

Environmental pollution with heavy metals (HMs) is of serious ecological and public health concern worldwide. Mining is one of the main sources of HMs and can impact the environment, species diversity, and human health. This study assessed the value of *Spilopelia senegalensis* as a biomonitor of environmental contamination with metal(loid)s caused by mining activities. *S. senegalensis* was collected from a gold mining site and a reference site, and metal(loid)s and biochemical parameters were determined. Lead, cadmium, mercury, vanadium, arsenic, copper, zinc, and iron were significantly increased in the liver, kidney, and lung of *S. senegalensis* from the mining site. Serum transaminases, alkaline phosphatase, creatinine, and urea were significantly elevated in *S. senegalensis* from the mining site. Lipid peroxidation and nitric oxide were increased, whereas glutathione and antioxidant enzymes were diminished in the liver and kidney of *S. senegalensis* from the mining site. In addition, multiple histological alterations were observed in the liver, kidney, and lung of *S. senegalensis*. In conclusion, mining activities provoke the accumulation of metal(loid)s, oxidative stress, and tissue injury in *S. senegalensis*. Therefore, *S. senegalensis* is a valuable biomonitor of environmental pollution caused by mining activities and could be utilized in epidemiological avian studies of human health.

## 1. Introduction

Bioindicators are living organisms utilized to appraise the health of the environment or natural ecosystem [1]. Different classes of indicator organisms may offer different responses to pollution; therefore, could they be used for biological monitoring [2]. Birds are widely distributed and occupy multiple habitat types and ecological niches. The presence of birds near the top of the food chain makes them sensitive to changes induced by environmental contaminants [3]. Given their well-treated classification and ease of detection in the environment [4], birds represent valuable biological indicators. In accordance, birds have been used as biomonitors to assess contamination of the environment with persistent organic pollutants, ecosystem health, biological effects of climate change, and different ecological processes [5,6,7,8]. The destructive effect of regular pollutants, such as inorganic fertilizers and pesticides, on bird populations has been explored in multiple studies [5,6,7,8]. Pollutants can also affect the breeding performance, as well as the survival, of birds [9]. In addition, exposure of birds to heavy metals (HMs) in the ecosystem is an issue of paramount importance. HMs produced through natural and anthropogenic ways can enter birds through direct inhalation, dermal contact, ingestion, and other routes [10]. In this context, pigeons have been suggested as valuable biomonitors and have been utilized in the assessment of atmospheric metal contamination in China [11].

Mining is the extraction of valuable materials and minerals from the earth. Mining practices create a negative environmental impact; therefore, most of the world’s nations have passed regulations to decrease the impact of pollution from mines [12,13,14]. Erosion, loss of biodiversity, dust, and contamination of water bodies and soil with HMs and other chemicals are among the environmental issues related to mining activities [15,16]. If not properly controlled, mining-related contamination with HMs can impact the health of the local population, as well as nearby communities [17]. The hazardous effects of HMs on the ecosystem, biodiversity, and health are related to their resistance to degradation and tendency for bioaccumulation and biomagnification in the food chain until reaching humans [18,19]. While some HMs have no specific metabolic role, others are essential for vital processes within the living cells, but are toxic at higher concentrations [20]. The toxic effects of HMs at concentrations beyond the physiological limits have been demonstrated both in vivo and in vitro [21,22]. Neurological disorders, osteoporosis, cancer, and other disorders are associated with the chronic exposure to HMs [23]. In rodents, exposure to HMs at mining and quarrying sites was associated with their accumulation in different tissues, resulting in liver, kidney, and lung injury [24,25].

Recently, we demonstrated the accumulation of HMs produced through gold mining activities in the soil and Arabian boxthorn and their negative impact on *Gerbillus nanus* in Saudi Arabia [24]. Given the importance of birds as biomonitors of environmental contamination, this study investigated the impact of gold mining activities in Riyadh (Saudi Arabia) on the laughing dove (*Spilopelia senegalensis*), with an emphasis on HM accumulation and tissue injury.

## 2. Materials and Methods

### 2.1. Collection and Processing of Samples

**Figure 1:**
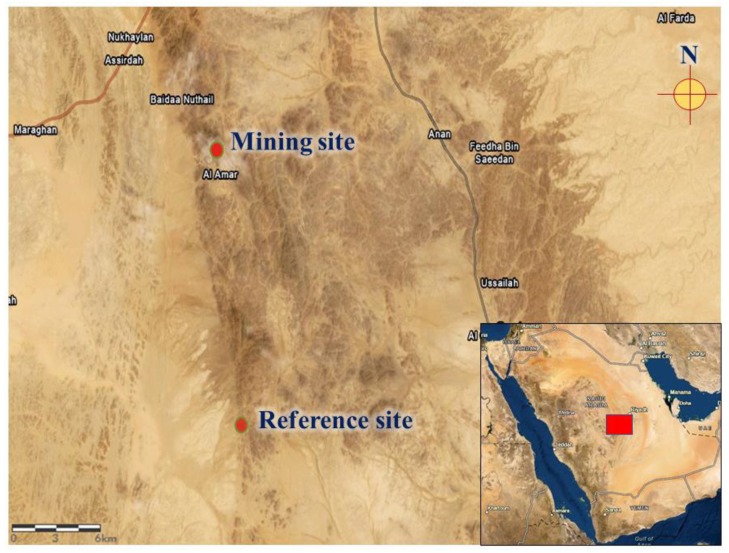
A map showing the location of the mining and reference sites.

Ten *S. senegalensis* were collected from the site of a gold mine located southwest of Al-Quway’iyah city, which is a large governorate located 165 km west of Riyadh Province (Saudi Arabia). The pigeons were collected within 0.5 km of the gold mine (45° 05′ E and 23° 47′ N) using bird traps. Ten other pigeons were collected from a reference site located 20 km away from the mine (45° 06′ E and 23° 36′ N). The reference site is a village with no industrial or mining activities (Figure 1).

The collected pigeons were transferred into the lab and immediately sacrificed, and blood was collected for serum separation. The pigeons were dissected, and the liver, kidneys, and lungs were collected, washed with ice-cold phosphate-buffered saline (PBS), and stored at −80 °C. Samples from the liver, kidneys, and lungs were collected on 10% neutral buffered formalin. Other samples were homogenized in PBS (10% *w*/*v*) for assaying lipid peroxidation (LPO), nitric oxide (NO), and the antioxidants reduced glutathione (GSH), superoxide dismutase (SOD), and catalase (CAT).

### 2.2. Assay of HMs and Arsenic (As)

The concentrations of lead (Pb), cadmium (Cd), mercury (Hg), vanadium (V), copper (Cu), zinc (Zn), iron (Fe), and As were determined in the liver, kidney, and lung samples of *S. senegalensis* using ELAN 9000 ICP-MS (Perkin Elmer Sciex Instruments, Concord, ON, Canada). Briefly, 2 mL nitric acid was added to 200 mg tissue sample in a clean digestion beaker. Following heating at 140 °C for 40 min, the digest was filtered, transferred to a clean tube, and the volume was brought to 10 mL using Ultrapure water. A blank digest was prepared in the same way. For calibration and quality control, standard references (Aristar grade, VWR International Ltd, Leicestershire, UK) were used. The linear rank of the methodology was assured by analyzing different standards for each element, and all standards were used in duplicate to determine the precision of the analysis. Ultrapure water was used to prepare blanks and calibration standards, and three replicate determinations were performed for each sample.

### 2.3. Histopathlogy

The tissue samples collected on 10% neutral buffered formalin were fixed for 48 h at 4 °C. The fixed samples were passed into a serial ascending grade of ethanol and xylene and embedded in paraffin wax. Then, 5-μm sections were cut and stained with hematoxylin and eosin (H&E) [26]. In brief, the sections were deparaffinized in three changes of xylene, rehydrated through a descending series of ethanol, and stained with hematoxylin. The slides were washed in tap water and then stained with eosin, followed by washing in tap water and rinsing in distilled water. The sections were dehydrated in ethanol, cleared in xylene, and mounted. The stained sections were examined using Coolscope Digital light Microscope (Nikon, Japan).

### 2.4. Assay of Liver and Kidney Function

Alanine transaminase (ALT), aspartate transaminase (AST), and alkaline phosphatase (ALP) are found within the hepatocytes and can be released into the bloodstream when the liver is damaged. Therefore, increased circulating levels of these enzymes indicates hepatocyte damage. Creatinine is a breakdown product of creatine phosphate in muscle, and urea is the major nitrogenous end product of protein and amino acid catabolism. Both creatinine and urea are filtered out of blood through the glomeruli, and are therefore commonly measured as indices of glomerular function [27,28]. To evaluate liver and kidney function in *S. senegalensis*, serum ALT, AST, ALP, creatinine, and urea were determined using kits purchased from Biomerieux (France), following the provided instructions.

### 2.5. Assay of LPO, NO, and Antioxidants

Oxidative stress has been highlighted as the main culprit behind the toxic action of most pollutants [29]. Therefore, assessment of oxidative stress markers and cellular antioxidants represents a potentially important indicator of the impact of environmental stressors on birds [30]. Increased production of reactive oxygen species (ROS) can provoke tissue injury by oxidizing lipids and proteins and depleting antioxidant defenses [31]. The impact of mining on the redox balance in *S. senegalensis* was evaluated by the determination of LPO, NO, and antioxidants. LPO was assayed as previously described by Preuss et al. [32], and NO was determined using Griess reagent following the method of Grisham et al. [33]. The antioxidant defenses GSH, SOD, and CAT were determined according to the methods of Beutler et al. [34], Marklund and Marklund [35], and Cohen et al. [36], respectively.

To normalize the results to protein, total protein content in the homogenates was assayed using Bradford assay [37].

### 2.6. Statistical Analysis

Data are expressed as means ± standard error of means (SEM). All statistical comparisons were performed by *t*-test using GraphPad Prism 7 (La Jolla, CA, USA). A *p*-value <0.05 was considered significant.

## 3. Results

### 3.1. Concentration of Metal(loid)s in the Liver, Kidney, and Lung of S. senegalensis

**Figure 2:**
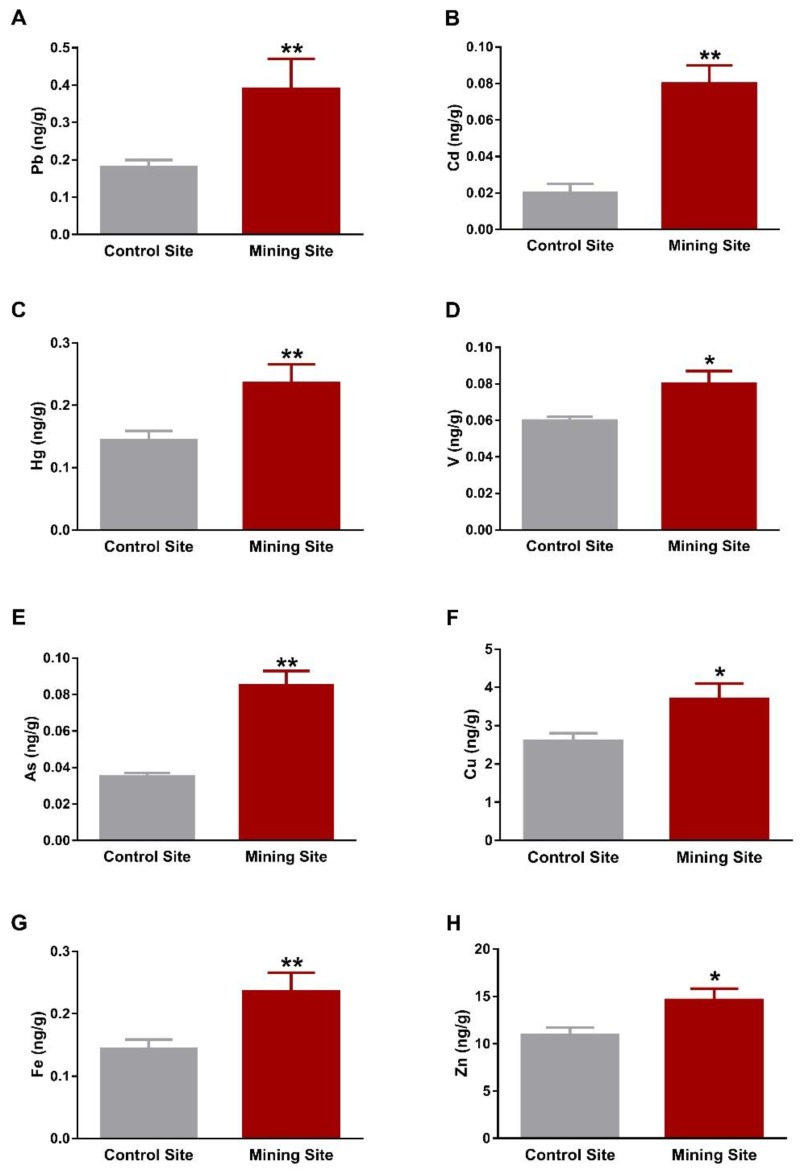
Concentrations of metal(loid)s in the liver of *S. senegalensis*. Pb (**A**), Cd (**B**), Hg (**C**), V (**D**), As (**E**), Cu (**F**), Fe (**G**), and Zn (**H**) were significantly increased in the liver of *S. senegalensis* from the mining site. Data are means ± standard error of means (SEM) (*n* = 10). * *p* < 0.05 and ** *p* < 0.01 versus the Control site.

Assessment of HM concentrations showed significant increase in Pb, Cd, and Hg in the liver of *S. senegalensis* collected from the mining site when compared with the control birds (*p* < 0.01), as depicted in Figure 2A–C. Similarly, the liver of *S. senegalensis* from the mining site showed elevated concentrations of V (*p* < 0.05), As (*p* < 0.01), Cu (*p* < 0.05), Fe (*p* < 0.01), and Zn (*p* < 0.05), as represented in Figure 2D–H.

**Figure 3:**
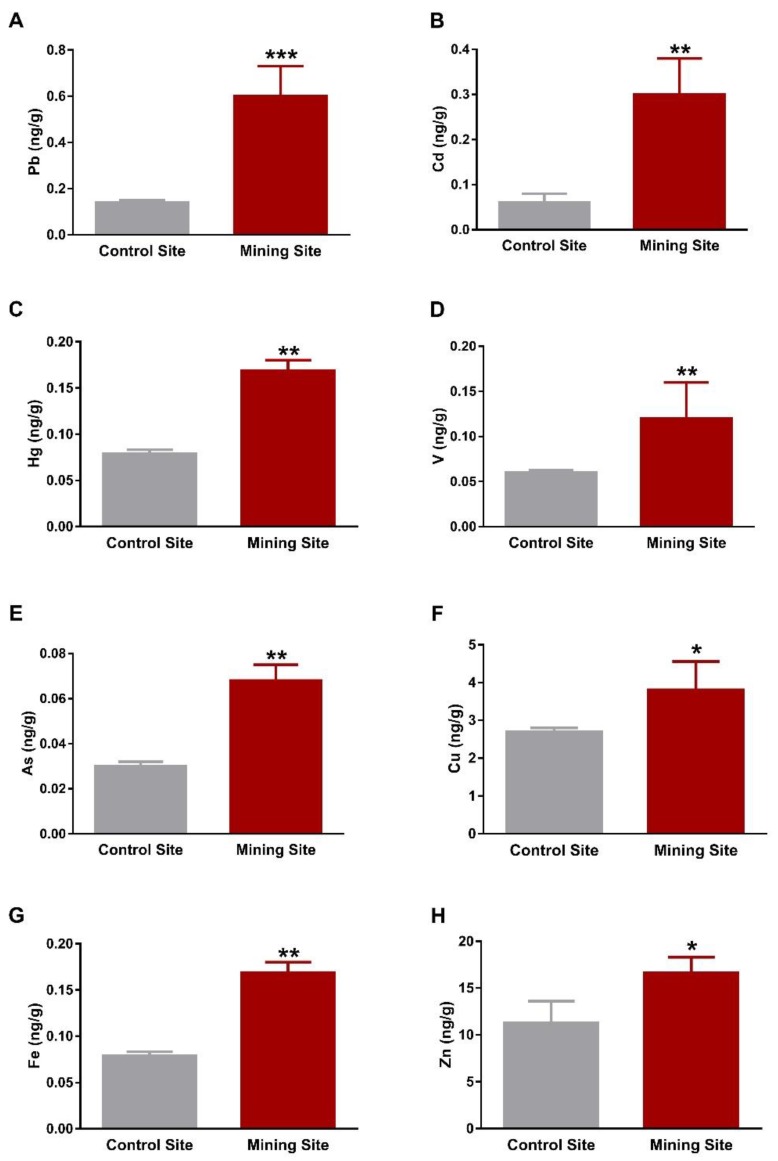
Concentrations of metal(loid)s in the kidney of *S. senegalensis*. Pb (**A**), Cd (**B**), Hg (**C**), V (**D**), As (**E**), Cu (**F**), Fe (**G**), and Zn (**H**) were significantly increased in the kidney of *S. senegalensis* from the mining site. Data are means ± SEM (*n* = 10). * *p* < 0.05, ** *p* < 0.01, and *** *p* < 0.001 versus the Control site.

**Figure 4:**
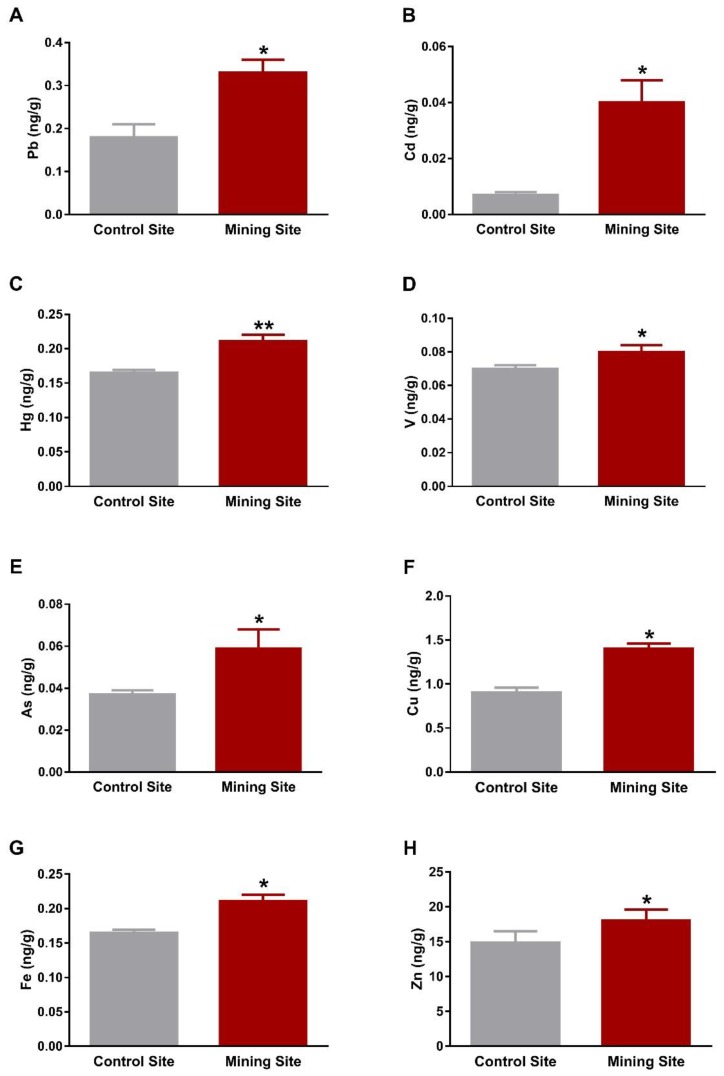
Concentrations of metal(loid)s in the lung of *S. senegalensis*. Pb (**A**), Cd (**B**), Hg (**C**), V (**D**), As (**E**), Cu (**F**), Fe (**G**), and Zn (**H**) were significantly increased in the lung of *S. senegalensis* from the mining site. Data are means ± SEM (*n* = 10). * *p* < 0.05 and ** *p* < 0.01 versus the Control site.

**Figure 5:**
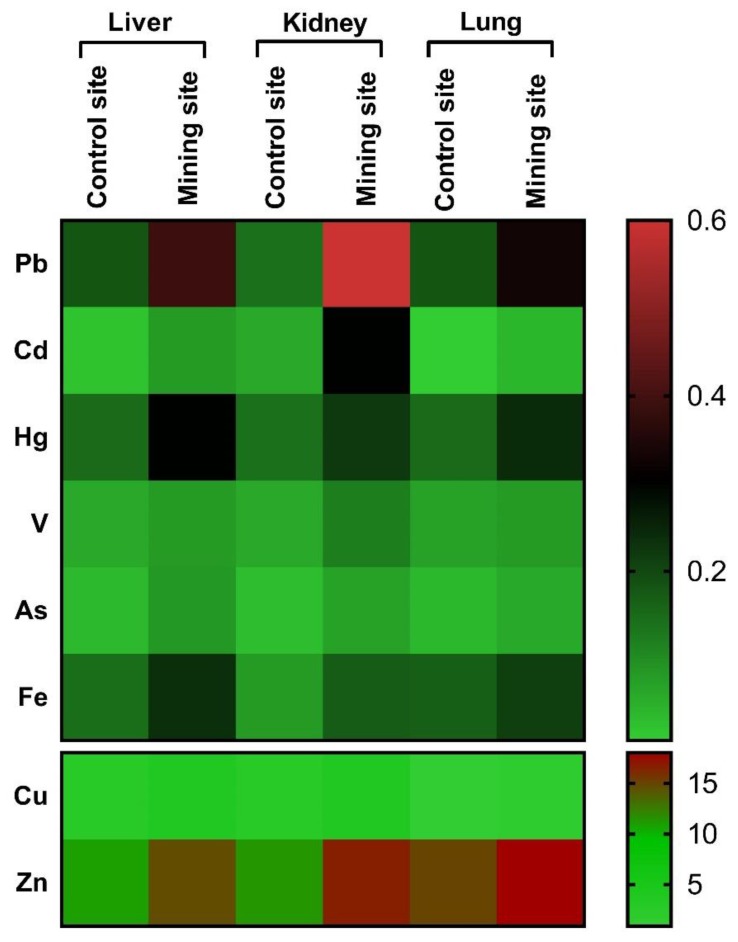
A heat map showing the differences between the concentrations of HMs in the liver, kidney, and lung of *S. senegalensis* collected from the mining and reference sites.

The kidney of *S. senegalensis* from the mining site exhibited significantly increased Pb (*p* < 0.001), Cd, Hg, V, As, Fe (*p* < 0.01), Cu, and Zn (*p* < 0.05) when compared with the control group, as shown in Figure 3A–H. Similarly, Pb, Cd, V, As, Cu, Fe, Zn (*p* < 0.05), and Hg (*p* < 0.01) concentrations were significantly increased in the lung of *S. senegalensis* collected from the mining site (Figure 4A–H). The differences between the concentrations of metal(loid)s in the liver, kidney, and lung of *S. senegalensis* collected from the mining and reference sites are summarized in Figure 5.

### 3.2. Effect of Mining on the Liver and Kidney Function of S. senegalensis

**Figure 6:**
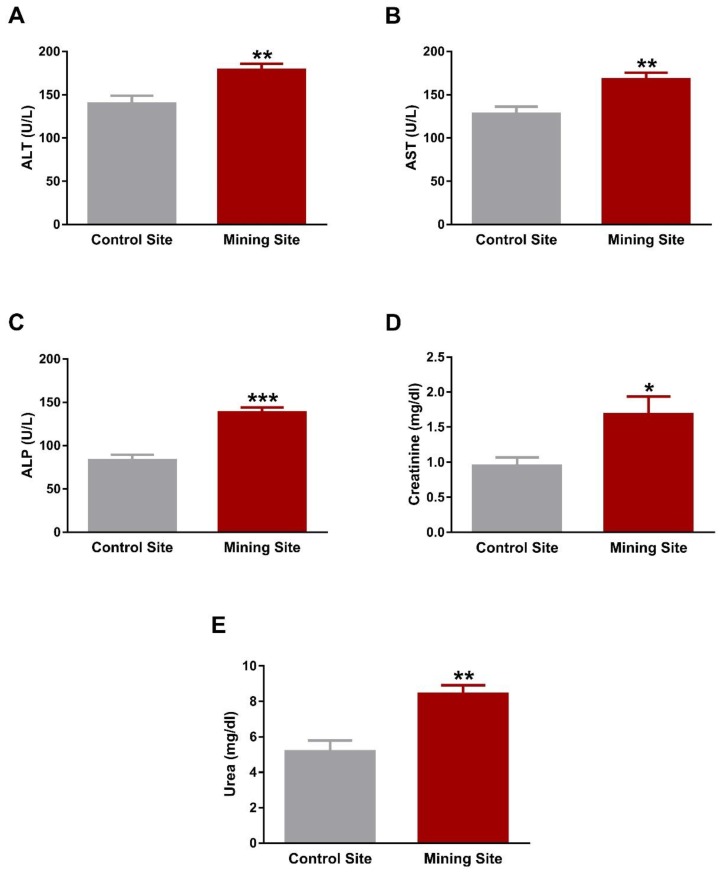
Liver and kidney function markers of *S. senegalensis*. ALT (**A**), AST (**B**), ALP (**C**), creatinine (**D**), and urea (**E**) were significantly elevated in serum of *S. senegalensis* from the mining site. Data are means ± SEM (*n* = 10). * *p* < 0.05, ** *p* < 0.01, and *** *p* < 0.001 versus the Control site.

The liver function markers ALT, AST, and ALP were significantly elevated in the serum of *S. senegalensis* collected from the mining site (*p* < 0.01; *p* < 0.01; *p* < 0.001) when compared with the reference site (Figure 6A–C). Serum levels of creatinine (Figure 6D) and urea (Figure 6E) showed a significant (*p* < 0.05; *p* < 0.01) increase in *S. senegalensis* collected from the mining site when compared with the reference pigeons.

### 3.3. Histopathological Changes Induced by Mining Activities in the Liver, Kidney, and Lung of S. senegalensis

**Figure 7:**
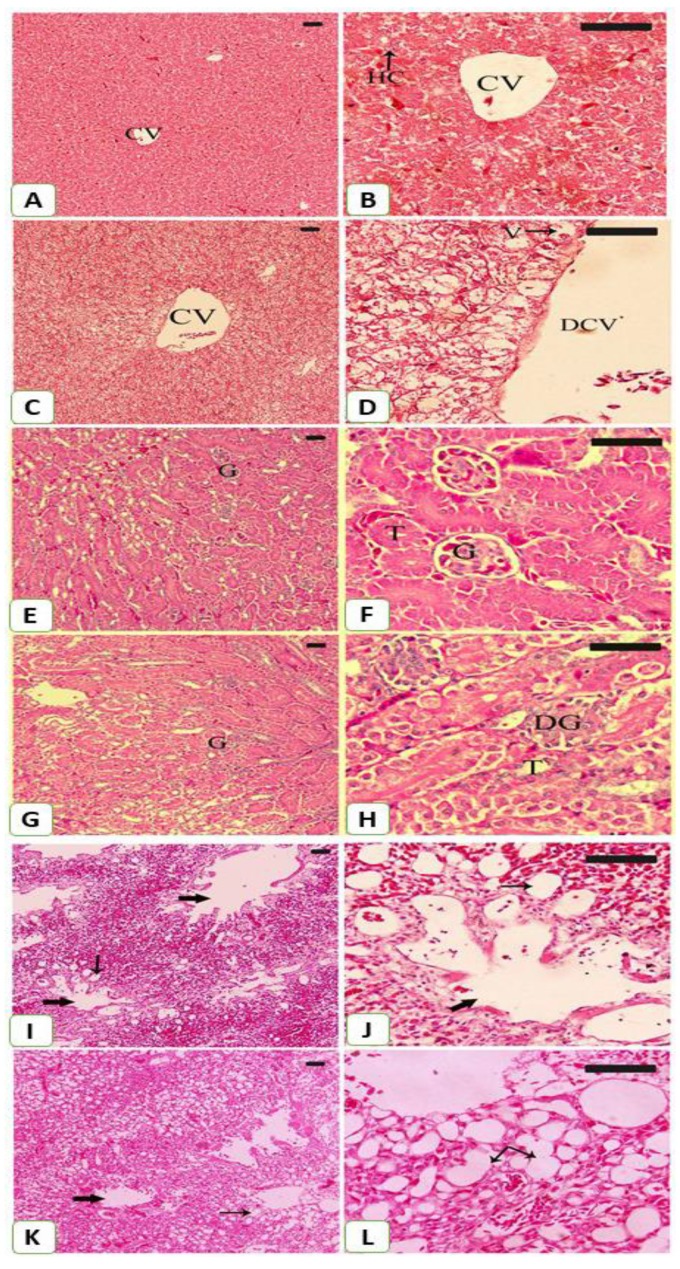
Photomicrographs of H&E-stained sections in the liver (**A**–**D**), kidney (**E**–**H**) and lung (**I**–**K**) of *S. senegalensis*. Pigeons from the reference site showed normal hepatocytes (HC), central vein (CV) (**A**,**B**), glomeruli (**G**), renal tubules (T) (**E**,**F**), alveoli and bronchioles (arrows) (**I**,**J**). Pigeons from the mining site showed dilated central vein (DCV), vacuolations (V) (**C**,**D**), degenerated glomeruli (DG), dilated renal tubules (T) (**G**,**H**), dilated alveoli and congested blood vessels (arrows) (**K**,**L**). (Scale bar = 100 µm).

The impact of mining on *S. senegalensis* was further evaluated by the histological findings (Figure 7). Examination of the H&E-stained liver section revealed normal structure of the hepatocytes and sinusoids in *S. senegalensis* from the reference site (Figure 7A,B). In contrast, the liver of *S. senegalensis* from the mining site showed histological alterations, including hepatocyte vacuolations and dilated central vein (Figure 7C,D).

The kidney sections of *S. senegalensis* at the control site revealed normal capsule, cortex, medulla, glomeruli, and renal tubules (Figure 7E,F), whereas glomerular degeneration was observed in *S. senegalensis* from the mining site (Figure 7G,H).

The lung of *S. senegalensis* from the control site showed normal structure of the bronchioles and alveoli (Figure 7I,J). In contrast, the lung of *S. senegalensis* from the mining site showed dilated alveoli and congested blood vessels (Figure 7K,L).

### 3.4. Mining Triggers Redox Imbalance in the Liver and Kidney of S. senegalensis

**Figure 8:**
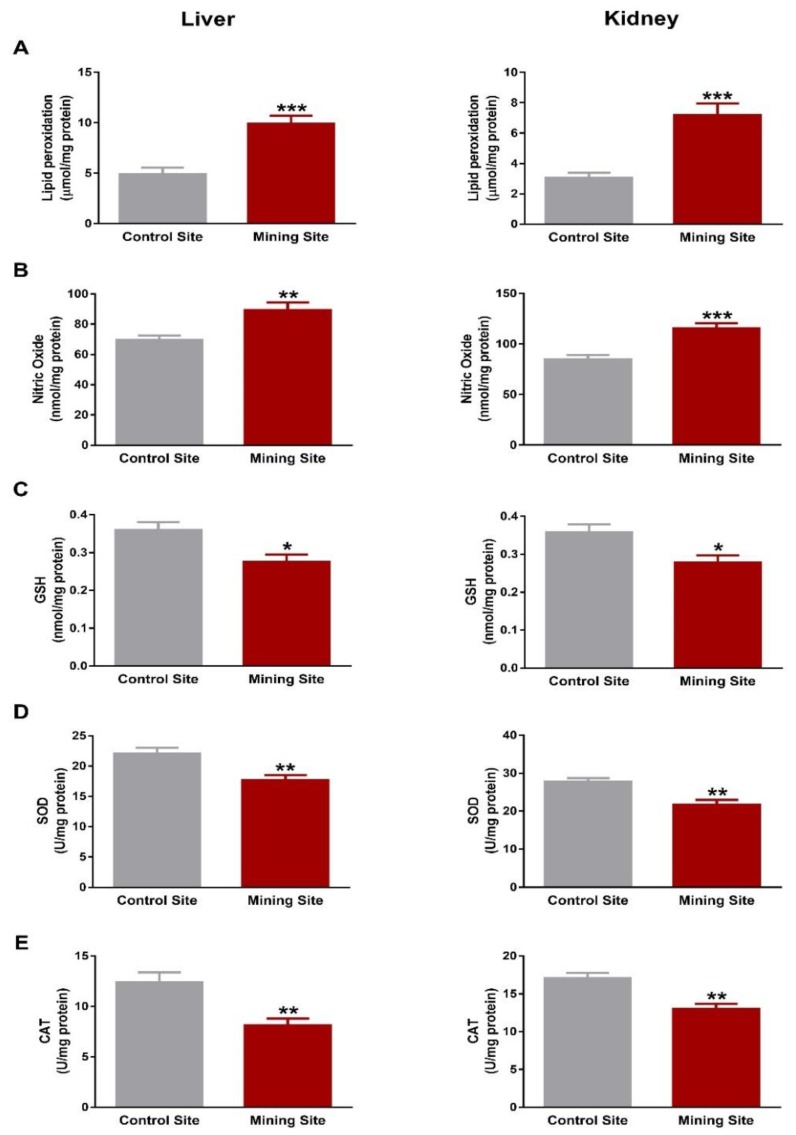
Oxidative stress markers and antioxidants in the liver and kidney of *S. senegalensis*. Hepatic and renal lipid peroxidation (**A**) and nitric oxide (**B**) were increased, and GSH (**C**), SOD (**D**), and CAT (**E**) were decreased in *S. senegalensis* from the mining site. Data are means ± SEM (*n* = 10). * *p* < 0.05, ** *p* < 0.01, and *** *p* < 0.001 versus the Control site.

To evaluate the impact of mining on the redox status in *S. senegalensis*, we determined LPO, NO, and antioxidants. *S. senegalensis* from the mining site exhibited a significant increase in liver and kidney LPO levels when compared with the reference sites birds (*p* < 0.001; Figure 8A). NO showed a significant increase in the liver (*p* < 0.01) and kidney (*p* < 0.001) of *S. senegalensis* from the mining site (Figure 8B).

Hepatic and renal GSH contents were decreased (*p* < 0.05) in *S. senegalensis* from the mining site, as represented in Figure 8C. Similarly, SOD (Figure 8D) and CAT (Figure 8E) were decreased significantly (*p* < 0.01) in the liver and kidney of *S. senegalensis* from the mining site when compared with the control site.

## 4. Discussion

Environmental contamination is one of the undesirable effects of mining, and different HMs, including Pb, Cd, Hg, Zn, and Cu, have been detected in the waste of mining [17]. Given their resistance to degradation, HMs can accumulate in the environment and cause negative impacts on the ecosystem and serious health problems [18,19,23]. Al-Quway’iyah, a big city in Riyadh (Saudi Arabia), is one of the sites of gold mining activities. We recently reported increased concentrations of HMs in the soil, plants, and different tissues of the Balochistan gerbil as a result of mining activities in Al-Quway’iyah [24]. The value of pigeons as biomonitors of environmental contamination has been recently demonstrated; therefore, we evaluated HM concentrations in different tissues of *S. senegalensis* at a gold mining site in Al-Quway’iyah, pointing to the resulted tissue damage and oxidative stress.

Analysis of metal(loid)s revealed an increase in Pb, Cd, Hg, V, Cu, Zn, Mn, Fe, and As concentrations in the liver, kidney, and lung of *S. senegalensis* at the site of gold mining activities. Accumulation of these metal(loid)s was associated with altered liver and kidney function, histological manifestations, and oxidative stress. *S. senegalensis* collected from the mining site showed increased serum ALT, AST, ALP, urea, and creatinine, demonstrating liver and kidney dysfunction. Aminotransferases and ALP are found inside the hepatocytes and their release into the circulation indicates hepatocyte damage. Creatinine and urea are commonly measured as indices of glomerular function [27,28]. Histological examination added support to the biochemical findings where hepatocyte vacuolations, dilated central vein, and glomerular degeneration were observed in the liver and kidney of *S. senegalensis* collected from the mining site. The tissue injury in *S. senegalensis* is directly connected to the increase in HMs and As concentrations, which are well-documented to pose a threat to different body organs [38].

Pb is a toxic HM with hazardous effects, ranging from mild physiological and biochemical disorders to severe pathological conditions. The exposure to Pb from agricultural and industrial activities is increasing [39]. The liver and kidney represent the main site for Pb deposition within the body [40] and this could explain the observed hepatic and renal tissue injury in *S. senegalensis* collected from the gold mining site. The toxicity of Pb is attributed to its ionic properties, where it can replace mono- and divalent cations in enzymes [41], and its ability to provoke excessive production of ROS and oxidative stress [42]. Increased ROS can trigger tissue injury through oxidizing lipids and proteins, inactivating antioxidant enzymes, and triggering DNA damage [31]. Accordingly, LPO and NO were increased and the antioxidants GSH, SOD, and CAT were decreased in both the liver and kidney of *S. senegalensis* collected from the site of mining activities. Besides liver and kidney injury, Pb was accumulated in the lungs of *S. senegalensis*, which showed histological alterations, an observation that was supported by our previous study showing the association between increased Pb concentration and tissue injury in the lungs of *Gerbillus nanus* collected from the same site [24]. Due to its highly toxic properties, Pb poisoning has been reported to cause the death of millions of birds each year [43].

Cd, even in trace quantities, causes physiological and health problems in birds, such as reduced growth performance and reproduction [10]. In birds, Cd caused severe necrosis in seminiferous tubules and damage all stages of germ cells, as reviewed by Marettová et al. [44]. It is a very toxic and undegradable HM that accumulates in plants due to its high transfer rate from the soil, and reaches birds and humans through the food chain [45,46,47]. Accordingly, our recent work showed an increase in Cd concentration in both soil and plants at the mining site in Al-Quway’iyah [24]. Herein, Cd accumulated in the lung, liver, and kidney of *S. senegalensis*, which showed tissue injury and dysfunction accompanied with oxidative stress. In this context, Cd has been demonstrated to trigger hepato- and nephrotoxicity, mainly via promoting oxidative stress [48,49]. Hydrogen peroxide (H_2_O_2_), which produces the highly toxic hydroxyl free radical through Fenton reaction, is produced by Cd within the body [50]. Upon entering the body, Cd is transported to the liver by albumin and forms complexes with metallothionine, thereby inducing liver injury. These complexes are transferred into the circulation and then accumulate and cause kidney injury [51,52]. Although cells are equipped with antioxidant enzymes which can counteract Cd-mediated H_2_O_2_ production, the activity of SOD and CAT was declined in *S. senegalensis* at the mining site as a result of Cd binding with the thiol groups of these enzymes [53]. The hepato- and nephrotoxic effects of Cd in birds have been previously reported. For instance, Cd induced hepatotoxicity [54] and nephrotoxicity [55] by triggering lipid peroxidation and histological alterations in *Gallus domesticus*. Furthermore, Cd accumulated in the lungs of *S. senegalensis* collected from the mining site, which showed dilated alveoli and congested blood vessels, indicating pulmonary toxicity. Lungs are one of the main routes of Cd entrance into the body [46], and diminished pulmonary function [56] and bronchial irritation and inflammation [57] are reported effects of Cd.

Mining is one of the main sources of Hg, which accumulates in soil, plants, and tissues of the rodents [24]. This study showed increased levels of Hg in the kidneys, liver, and lungs of *S. senegalensis* collected from the mining site. Hg accumulation played a role in the nephro-, hepato-, and pulmonary toxicity observed in *S. senegalensis*. Hg has been reported to decrease fertility, egg weight, and embryonic growth, and induce kidney lesions in wild birds [5,8]. Both Hg and methylmercury trigger LPO and apoptosis, and therefore, cause nephro- and hepatotoxicity [58,59,60]. This study conferred new information that the hazardous effect of mining activities on birds is associated with increased concentrations of Hg.

V and As are environmental pollutants produced through industrial activities, including mining, and can accumulate in plants and soil and affect wild animals [24]. Here, *S. senegalensis* collected from Al-Quway’iyah mining site showed increased concentrations of V and As, which are known to exert toxic effects. V and its pentoxide caused occupational toxicity, chronic productive cough, and bronchial inflammation when inhaled [61,62], and triggered liver injury in rats [63]. As was found to be teratogenic in brooding birds and damage chromosomes in bone marrow cells of birds [64,65]. As genotoxicity has been postulated to be linked to excess ROS production, DNA damage, activation of apoptosis signaling, and replacing metal ions in enzymes and proteins [66]. The toxicity of As is associated with the formation of inorganic highly toxic and carcinogenic intermediates [67]. In birds, As compounds increased the incidence of renal tumors [64,65]. Moreover, exposure to As has been associated with liver, kidney, and lung injury in experimental animals [68,69,70]. Therefore, exposure of *S. senegalensis* to V and As at the mining site resulted in tissue injury and oxidative stress.

Fe, Zn, and Cu were also increased in the liver, kidney, and lung of *S. senegalensis* collected from the mining site and could be associated with the observed tissue injury, dysfunction, and oxidative stress. Cu is potentially toxic as it exists in oxidized [Cu(II)] state in the environment and ROS are generated during its transition into the reduced form [71]. Fe and Zn are essential for many cell functions; however, they are toxic at concentrations beyond the physiological limits. Increased Fe is associated with hepatotoxicity [72] and nephrotoxicity [73], and high Zn concentrations can replace essential elements or interact with the sulfhydryl groups of multiple proteins [74]. Zn concentrations in the liver, kidney, and lung of *S. senegalensis* were found to be higher than other HMs. Although it is required for the function of a large number of enzymes and transcription factors within the body, high Zn concentration can be harmful [75]. Therefore, accumulation of Fe, Zn, and Cu can induce oxidative stress and tissue injury.

The accumulation of HMs in different tissues of pigeons collected from contaminated regions was reported in previous studies; however, the impact of gold mining on *S. senegalensis* has not been reported, at least not in Saudi Arabia. The feral pigeons (*Columba livia*) collected from a ferronickel smelter courtyard in Drenas (Kosovo) exhibited significantly increased concentrations of Pb, Cd, Zn, Cu, and Ni in the liver, kidney, and other tissues when compared with pigeons collected from a control site [76]. Feral pigeons from the same area (Drenas, Kosovo) showed liver dysfunction and accumulation of HMs in different tissues [77]. Hence, the feral pigeon has been suggested as a biomonitoring organism for the evaluation of environmental pollution caused by ferronickel industry [77]. In addition, the accumulation of Pb and Cd in tissues of feral pigeons collected near central London has been demonstrated [78]. The findings of this study suggest the value of the feral pigeon to monitor urban Pb contamination and as a model for chronic Pb toxicity [78].

## 5. Conclusions

The current findings introduce information on the value of *S. senegalensis* as a biomonitor of environmental contamination caused by mining activities. The results showed an increase in the concentrations of Pb, Cd, Hg, V, As, Zn, Fe, and Cu, histopathological alterations, increased lipid peroxidation, and decreased antioxidant defenses in different tissues of *S. senegalensis* collected from the mining site. These data closely reflect the differences in HM concentrations between the mining and control sites and suggest that *S. senegalensis* provide valuable data for evaluating the impact of environmental pollutants. Moreover, this study might present the scientific basis for employing *S. senegalensis* in epidemiological avian studies of human health.
